# Metaproteomics-guided selection of targeted enzymes for bioprospecting of mixed microbial communities

**DOI:** 10.1186/s13068-017-0815-z

**Published:** 2017-05-16

**Authors:** Jutta Speda, Bengt-Harald Jonsson, Uno Carlsson, Martin Karlsson

**Affiliations:** 1 Molecular Biotechnology, Dept. of Physics, Chemistry and Biology, Linköping University, 581 83 Linköping, Sweden; 2 Biochemistry, Dept. of Physics, Chemistry and Biology, Linköping University, 58183 Linköping, Sweden; 3 InZymes Biotech AB, Gjuterigatan 1B, 582 73 Linköping, Sweden

**Keywords:** Biofuel, Metaproteome, Bioprospecting, Microbial community, Enzyme discovery, Induction, Extracellular, Cellulase, Biogas, Protein inference

## Abstract

**Background:**

Hitherto, the main goal of metaproteomic analyses has been to characterize the functional role of particular microorganisms in the microbial ecology of various microbial communities. Recently, it has been suggested that metaproteomics could be used for bioprospecting microbial communities to query for the most active enzymes to improve the selection process of industrially relevant enzymes. In the present study, to reduce the complexity of metaproteomic samples for targeted bioprospecting of novel enzymes, a microbial community capable of producing cellulases was maintained on a chemically defined medium in an enzyme suppressed metabolic steady state. From this state, it was possible to specifically and distinctively induce the desired cellulolytic activity. The extracellular fraction of the protein complement of the induced sample could thereby be purified and compared to a non-induced sample of the same community by differential gel electrophoresis to discriminate between constitutively expressed proteins and proteins upregulated in response to the inducing substance.

**Results:**

Using the applied approach, downstream analysis by mass spectrometry could be limited to only proteins recognized as upregulated in the cellulase-induced sample. Of 39 selected proteins, the majority were found to be linked to the need to degrade, take up, and metabolize cellulose. In addition, 28 (72%) of the proteins were non-cytosolic and 17 (44%) were annotated as carbohydrate-active enzymes. The results demonstrated both the applicability of the proposed approach for identifying extracellular proteins and guiding the selection of proteins toward those specifically upregulated and targeted by the enzyme inducing substance. Further, because identification of interesting proteins was based on the regulation of enzyme expression in response to a need to hydrolyze and utilize a specific substance, other unexpected enzyme activities were able to be identified.

**Conclusions:**

The described approach created the conditions necessary to be able to select relevant extracellular enzymes that were extracted from the enzyme-induced microbial community. However, for the purpose of bioprospecting for enzymes to clone, produce, and characterize for practical applications, it was concluded that identification against public databases was not sufficient to identify the correct gene or protein sequence for cloning of the identified novel enzymes.

## Background

The discovery and identification of novel biocatalysts is an important goal in enzyme research and industrial biotechnology. To date, most enzymes have been discovered by using classical, selective microbial screening methods. The selected organisms are then individually isolated and grown as pure cultures originating from a single colony with the desired enzyme activity. However, this selective isolation limits the number of possible enzyme source candidates because only a minute fraction of all microorganisms can be obtained as pure cultures in the laboratory [1, 2]. In nature, microorganisms exist in microbial communities and are often reliant on the syntrophic benefits that are afforded by the different populations of the community. Hence, it has been estimated that less than 1% of all microorganisms can be obtained in pure cultures using standard methods, naturally limiting access to novel enzymes [3]. Consequently, if only pure cultured microorganisms are considered for enzyme discovery, some 99% of the potential sources for novel enzymes will be overlooked. For this reason, the development of systems biology metaomics approaches is instrumental. In metaomics, the microbial communities are considered to represent a “metaorganism.” This is a valid understanding of microbial communities because the majority of microorganisms are apparently not able to survive without the support of the complete body of microorganisms in a given habitat. Therefore, to include the overlooked 99% of microorganisms in bioprospecting efforts, pure culture-independent metaomics techniques are needed.

Rapid technology development in mass spectrometry and next generation sequencing (NGS) has enabled large-scale metaproteomic [4] and metagenomic [5] studies of microbial communities without the need for pure culturing. However, these approaches have so far mainly been used to understand the interactions of microbes in ecosystems or certain habitats [6–8]. Furthermore, although the methods in theory give access to the entire protein complement and the entire genetic potential of microbial communities, both metaproteomics and metagenomics are largely non-targeting and individually limited in their use for targeted bioprospecting. By the use of metagenomics, it is possible to find the genetic potential for sought after enzymes. However, even if genes for a certain enzymatic activity are identified, no information is obtained on whether the enzyme is produced under certain circumstances or the applicability of the enzyme, which is governed by its properties with regard to stability, efficiency, and specificity [9]. Recently, to improve the selection process of applicable enzymes, it has been suggested that microbial consortia should be analyzed by integrating different omics-techniques, such as metagenomics and metaproteomics [10, 11]. In bioprospecting efforts for discovery of novel enzymes, this would provide a direct link between the actually expressed and utilized enzyme and the corresponding gene. However, there have been no suggestions on how this can be achieved by the use of metaproteomics.

The term prospecting implies that an area of interest holds something of value worth looking for. Thus, bioprospecting is not an activity based on chance but is a targeted search for that value in a defined setting. One obvious value and application of bioprospecting in microbial communities is, thus, the search for novel enzymes that have activities and properties that make them suitable for industrial processes. While the incentive for bioprospecting for novel enzymes in microbial communities is clear, how to achieve targeted bioprospecting for novel enzymes by metaproteomics is less obvious given the magnitude of the problem and the limitations of available techniques, e.g., sample preparation [12, 13]. Metaproteomics is defined as “the large-scale characterization of the entire protein complement of environmental microbiota at a given point in time” [4]. The key words “at a given point in time” mean that the protein expression profile at several time points can be compared to understand the dynamics in protein expression. Thus, metaproteomics allows short-term interventional studies of protein expression, as opposed to more observational studies of the static metagenome. However, since it is the protein expression dynamics of microbial communities that is studied, a huge number of changes in protein expression can be expected. In this context, it should be noted that metagenomics and metaproteomics of microbial communities differ profoundly from, e.g., genomics and proteomics of multicellular higher organisms. For instance, in humans, all the approx. 200 different cell types contain the same genome of approx. 21,000 genes, of which 60–70% are expressed at any time [14]. In contrast, the different cells making up the body of microorganisms in a mixed microbial community do *not* contain the same genome. Of the 4191 predicted genes present in the complete genome of *E. coli,* 2800, or approx. 2/3 of all genes, are estimated to be expressed at any time [15]. If these numbers are applied to a microbial community of e.g., 200 different species (cell types), each with an average genome of 4200 genes, there would possibly be 560,000 different proteins present at any time, many of which are more or less homologous. Thus, if the change in protein expression between two different conditions is investigated, the expression of any or all of potentially 840,000 different proteins could change. It has been estimated that for a meta-proteome derived from a complex microbial communities, ≪1% of the entire protein complement can be resolved [16]. Thus, to understand the dynamics in response to e.g., an external stimulus, close control must be exercised to ensure that the response is not due to too many influences. Such close control is unlikely to be possible in natural environments with fluctuating temperatures, pH, organic load, nutrients, etc. Because of this complexity, the value of more defined systems has recently been emphasized [17]. Furthermore, in industrial biotechnology, the vast majority of enzymes used are hydrolytic enzymes [18–20]. Many of these enzymes are normally secreted to the extracellular environment by microorganisms in order to hydrolyze and make available nutrients from more complex biomolecular substances. However, it is usually not possible to accurately analyze these extracellular proteins in samples from natural environments by metaproteomics because these proteins cannot easily and reproducibly be separated and concentrated from the extracellular matrix. Therefore, the enzymes most interesting for industrial biotechnology are largely unattainable for studies by metaproteomics. Because of these issues, the vast majority of all metaproteomic studies of microbial communities are performed on only the intracellular fraction of the metaproteome, although there are exceptions in studies of more defined systems [21]. In addition, many studies are performed on a single state at a single time point, simply providing a snapshot of the metaproteome without exploiting the dynamic changes in protein expression that define metaproteomics.

To progress metaproteomics for targeted bioprospecting of novel enzymes in microbial communities and provide solutions to the problems of complexity, targeting and sample preparation for extracellular proteins, an approach using a microbial community in a constructed environment was earlier established [22]. In this earlier work, a complete methanogenic microbial community was maintained under controlled conditions using a chemically defined medium of simple nutrients. By this approach, a microbial community at metabolic steady state was obtained in which expression of extracellular hydrolytic enzymes was suppressed, thus providing baseline expression of extracellular proteins. From this enzyme suppressed metabolic steady state, it was found that the desired hydrolytic enzyme activity could be induced at will by replacing the simple nutrients with complex counterparts, e.g., by exchanging glucose for cellulose to induce cellulolytic activity. In addition, it was further found that the extracellular enzymes could be purified to provide 2-D gels of high quality, allowing detection of also less abundant proteins [23]. Recently, the same system was used to produce a mixture of cellulolytic enzymes that were shown to increase the hydrolysis rate of lignocellulosic biomass in biogas batch experiments [24]. Efficient degradation of lignocellulosic biomass is a key step in the production of biogas and is dependent on the interaction of different classes of enzymes, such as exoglucanases, endoglucanases, and β-glucosidases [25]. Thus, using differential gel electrophoresis, it should be possible to compare the extracellular metaproteome of a well-defined cellulase-induced state with a complementary and equally well-defined non-induced state to discriminate between proteins induced in response to cellulose and proteins constitutively expressed in the enzyme suppressed state. Downstream analysis by mass spectrometry may then be limited to only those proteins identified as upregulated in response to the need to hydrolyze and utilize cellulose as a nutrient source, thereby circumventing much of the complexity problem.

To find out whether this hypothesis was valid, the enzyme induction experiment was repeated and the enzyme expression pattern of a cellulase induced state was compared to a non-induced state with the aim of answering the following questions: (i) can the protein expression pattern of the enzyme-induced state be compared to a pre- or non-induced state to identify enzymes that become upregulated upon induction, (ii) can the enzymes identified as upregulated be confirmed to be predominantly actively secreted extracellular proteins, (iii) can these proteins be linked to the inducing substance in order to confirm preferential selection of the targeted cellulolytic enzymes against a large background of other proteins, and finally, (iv) can the approach be used for targeted enzyme discovery by bioprospecting in microbial communities in order to clone, produce, and characterize identified enzymes.

## Methods

### Induction and monitoring of cellulase activity

The microbial methanogenic community maintained in an enzyme suppressed and metabolic steady state used for the induction of cellulolytic enzymes has earlier been described in detail [22]. For the induction of cellulolytic enzymes, an aliquot of 1 l of the microbial methanogenic community was collected from the bioreactor at the end of a feeding cycle (24 h after previous feeding). The community culture was transferred to N_2_-purged centrifugal bottles and centrifuged for 30 min at 9000×*g* and 37 °C. After centrifugation, the supernatant was discarded and the cell pellet was resuspended in 800 ml of preheated (37 °C) buffer/mineral solution of the same composition as used for the chemically defined medium, i.e., buffer/mineral solution with no nutrients. For differential batch culturing, the community cell suspension was divided into two samples of 400 ml each and transferred to N_2_-flushed 500-ml glass bottles, which were then sealed with a rubber stopper and an aluminum cap. To these two suspensions, 400 µl each of vitamin stock, trace element, and ultra-trace element stock solution were added to replicate conditions in the bioreactor. The reference batch sample was fed the same carbon sources as the microbial community at steady state in the bioreactor. In contrast, for the cellulase induction batch sample, 1 g of cut filter paper (Whatman no1, Whatman Ltd, USA) was added in a closed nylon mesh bag as the sole carbon source. To prevent degradation of any expressed extracellular proteins, 1 tablet of protease inhibitor (cOmplete, Roche Diagnostics, Mannheim, Germany) was added to each batch bottle. The samples were incubated at 37 °C for 11 days without agitation. During that time, the gas pressure, methane content, pH, and cellulase activity were repeatedly analyzed. Thus, both samples were treated identically up until separation into different batch bottles. Afterwards, the washed reference sample was subjected to the same conditions and same source of simple nutrients as in the bioreactor, whereas the cellulase induction sample had the same conditions but a complex source of nutrients in the form of filter paper.

Cellulase activity was monitored using a fluorescent cellulase assay kit [26] (Marker Gene Technologies Inc. Eugene, USA) as earlier described [22]. For easier comparison of the cellulase activity during the duration of the experiment, the amount of free resorufin after 30-min incubation in the assay was used to calculate the activity and as a comparison during different days. For calculation of the cellulase activity in U/ml, a resorufin standard curve was prepared using the same buffer solutions and instrumental settings as used in the assay. The pH of the batch supernatant was determined using pH indicator strips (Neutralit pH 5.0–10.0, Merck KGaA, Darmstadt, Germany) following the manufacturer’s instructions.

### Biogas and biomethane production

The pressure in the batch bottles was measured daily with a pressure gauge (Testo 312-3, Testo AG, Germany) immediately prior to collecting liquid samples from the batch bottles. After pressure reading and sampling, the gas pressure was released and allowed to equalize to atmospheric pressure. The amount of produced gas and methane was calculated by considering the volume of headspace in the bottles, which due to sample extraction, changed over time. All values were normalized to SI standard conditions and presented as accumulated biogas production. After 8 days of incubation, a 2.5-ml gas sample was collected from each batch bottle for analysis of the methane content by gas chromatography. Methane concentration was determined using a GC-FID instrument (gas chromatography—flame ionization detector). A sample of 100 µl was injected via a gas loop to a Porapak T80/100 mesh column (Perkin-Elmer, Waltham, USA) using N_2_ as a carrier gas at 80 °C and a flow rate of 44 ml/min. The results were compared to a standard curve of methane readings.

### Extraction of extracellular proteins

At selected time points, 100 ml of culture supernatant was collected and concentrated to a final volume of 10 ml using a Spin-X 5 kDa PES (polyethersulfone) centrifugal filter (Corning, USA). 10 ml of cooled extraction buffer (2× [50 mM Tris–HCl, pH 8,5, 5 mM EDTA, 100 mM KCl, 1% (w/v) DTT, 30% (w/v) sucrose]), containing the cOmplete protease inhibitor mix (Roche) were added to the 10-ml concentrated sample [24]. After vortexing, protein samples were extracted with 10 ml Tris-buffered phenol, pH 8.0 (Sigma, Germany). After phase separation, the phenol phase was transferred to a new tube and again extracted with extraction buffer. The phenol phase was pipetted into a new tube and proteins were precipitated by overnight incubation with 0.1 M ammonium acetate in methanol at −20 °C. The precipitated proteins were collected by centrifugation and the pellet was washed three times with ice-cold acetone before air-drying. The protein pellet was dissolved in 500 µl 2-D DIGE buffer (2 M thiourea, 7 M urea, 4% (w/v) CHAPS, 30 mM Tris–HCl pH 8.5) and the protein concentration was determined using the 2D Quant kit (GE Healthcare).

### 2-D differential gel electrophoresis (DIGE)

50 µg of each sample was labeled with a separate CyDye before loading onto a gel. A second 2-D DIGE gel replicate was prepared in a similar way but with switched dyes to compensate for possible altered dye affinity. IPG strips (range 4–7, Bio-Rad) were rehydrated overnight at 20 °C with rehydration buffer (2 M thiourea, 7 M urea, 1% (w/v) CHAPS, 0.4% DTT and 0.5% carrier ampholytes, Bio-Rad). Samples were then loaded via an anodic cup to the IPG strips for 6 h at 200 V prior to focusing on a Protean IEF cell (Bio-Rad). The following voltage ramp protocol was used on the Protean IEF cell (Bio-Rad): 30 min 250 V rapid voltage ramping, 1 h 8000 V slow voltage ramping, rapid voltage ramping to 20 kVh, followed by rapid ramping to 50 V for 20 h (holding step). The IPG strips were refocused for 30 min at 8000 V prior to 2nd dimension analysis and equilibrated for 15 min in equilibration base buffer I (6 M urea, 30% (w/v) glycerol, 2% (w/v) SDS in 0.05 M Tris–HCl buffer (pH 8.8, 1% DTT), followed by 15 min in equilibration base buffer II (6 M urea, 30% (w/v) glycerol, 2% (w/v) SDS in 0.05 M Tris–HCl buffer pH 8.8, 4% iodoacetamide). The second dimension was run on a Criterion Cell system (Bio-Rad) using Bio-Rad’s Criterion 12% Bis–Tris gels in a MOPS buffer system at 50 V for 30 min, followed by 200 V for 50 min. Images were captured on a Versadoc CCD camera system (Bio-Rad, USA) with an exposure time of 10 s for each CyDye fluorophore.

A preparative gel was run for spot picking. 150 µg of the cellulase-active sample dissolved in thiourea/urea lysis buffer (2 M thiourea, 7 M urea, 1% (w/v) CHAPS, 0.4% DTT and 0.5% carrier ampholytes, Bio-Rad) was applied by anodic cup loading to a rehydrated IPG strip (pH 4–7) and separated using the same methods as applied above. After 2-DE, the gel was fixed for 2 h in fixation solution (40% ethanol, 10% acetic acid) and stained overnight with colloidal Coomassie G-250 Blue solution (0.1% Coomassie G-250, 10% (NH_4_)_2_SO_4_, 3% H_3_PO_4_ in 20% ethanol) using a protocol modified from Neuhoff et al. [27] under gentle agitation at RT. After washing and destaining with Milli-Q H_2_O, an image was captured for documentation and manual identification of the upregulated spots identified by the 2-DE DIGE experiment.

### Image analysis and selection of upregulated proteins

DIGE gels were analyzed with the PDQuest advanced image analysis software by Bio-Rad, USA. To minimize manual input and bias, standard settings were used when possible. The gels were analyzed according to the manufacturer’s instructions and guidelines for DIGE analysis. For image analysis, a master gel was generated from all six samples, including the dye switched ones. The protein expression patterns of each sample and dye variant were then compared to the master gel to assess how well the individual samples matched to it before comparing the intensity in each spot. All protein spots identified as new or at least 2-times upregulated for the cellulase-induced sample compared to the non-induced sample were considered for further analysis. Protein spots selected as upregulated by image analysis and visible to the naked eye on the preparative Coomassie Blue stained gel were manually picked in a sterile hood and digested with modified trypsin (Promega, The Netherlands) following standard protocols.

### Nano LC–MS/MS and data analysis

The obtained peptide mixtures were applied to an EASY-nanoLC II system (Bruker Daltonics, Bremen, Germany) coupled online to a LTQ (linear trap quadrupole) Orbitrap Velos Pro hybrid mass spectrometer (Thermo Fisher Scientific). Peptides were separated during 60 min by reverse phase chromatography on a 20 mm × 100 µm C18 pre-column followed by a 100 mm × 75 µm C18 analytical column (particle size 5 µm, NanoSeparations, Nieuwkoop, Netherlands) at a flow rate of 300 nL/min. A gradient of 0.1% formic acid in water (A) and 0.1% formic acid in acetonitrile (B) was distributed as follows: starting with 2% B; linear gradient 2–40% B in 0–40 min; 40–90% B in 40–60 min. Mass spectra were acquired in positive profile mode by FTMS (Fourier transform mass spectrometry) at a resolution of 30,000 (at *m*/*z* 400). The top 20 most intense multiply charged ions were selected with an isolation window of 2.0 and fragmented in the linear ion-trap by collision-induced dissociation (CID) using a normalized collision energy of 30. Dynamic exclusion of sequenced peptides for 60 s and charge state filtering disqualifying singly charged peptides were activated and predictive AGC (automatic gain control) was enabled. The centroid mode was used for CID MS/MS (collision-induced MS/MS).

### Mass database and homology search

Thermo Excalibur.RAW files were analyzed using the PEAKS 6 software engine (Bioinformatics solutions Inc.) for mass spectrometry data. For data refinement, default settings were used that automatically corrected the mass of the precursor ion. A PEAKS DB database search was performed [28] using an error tolerance of 15.0 ppm for the monoisotopic precursor mass, a fragment ion mass of 0.5 Da and trypsin as the enzyme with one non-specific cleavage at the end of the peptide and maximum two missed cleavages per peptide. As posttranslational modifications (PTMs), carbamidomethylation of cysteine was set as fixed, whereas oxidation of methionine and acetylation of the N-terminus were assumed as variable modifications. The databases used for the search were the NCBI non-redundant (nr) database (downloaded 2016-05-03) with taxonomy filters for archaea and bacteria. For the NCBI nr database search, unspecified PTMs were analyzed by running the PEAKS PTM algorithm. The false discovery rate (FDR) was estimated with decoy fusion and the homology match algorithm SPIDER was performed on spectra with a de novo average local confidence score (ALC% score) greater than 30% using the same PTM settings as in the PEAKS DB database search. The FDR was set to 1% to minimize false positive hits.

The PEAKS 6.0 search engine was used for producing de novo sequence tags. Thereafter, PEAKS DB [28] was used for searches in the NCBI nr database and the uniprot polysaccharide degradation database. In addition, the SPIDER algorithm of PEAKS was used for homology searches [29, 30]. The results from the database search on NCBI nr were limited to the taxa of bacteria and archaea and the MS hits were filtered for molecular weights as matched to the preparative 2D-gel. As the proteins chosen for analysis were upregulated upon induction with cellulose and the sample was withdrawn from a sample with high cellulase activity, the analysis was restricted to proteins involved in carbohydrate degradation only. Protein hits were assigned to the 2-D gel by filtering for a corresponding or higher molecular weight because small spots on a 2D gel may be fragments of larger proteins. Enzymes involved in cellulose degradation were considered as identified when at least 2 unique peptides for a FDR of 1%, a protein score (−10lgP) ≥20 and de novo ALC score ≥50%, were confirmed and involvement in carbohydrate degradation could be verified. The molecular function was either determined from the databases or by using the InterPro protein sequence analysis classification [31]. The cellular location of the respective protein hit was analyzed by retrieving the protein sequence from NCBI by the GI number and running a transmembrane topology and signal peptide predictor using Phobius [32].

## Results

### Cellulase induction and protein sample preparation

**Fig. 1:**
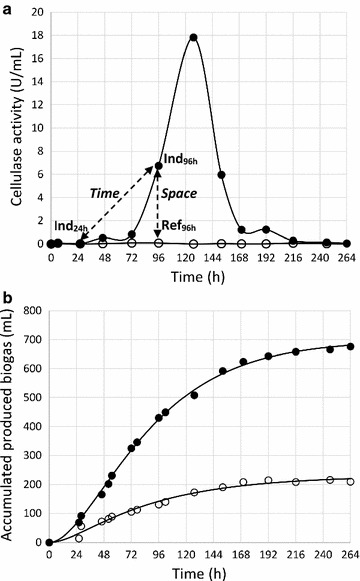
**a** Cellulase activity and biogas production in induced and non-induced samples. **a** Cellulase activity in the induced sample (*filled circle*) and the non-induced reference sample with the original chemically defined medium (*open circle*). *Dotted arrows* indicate samples compared over time in the induced sample, and over space against the non-induced reference sample. **b** Gas production in the two samples over the same time period, indicating viable microbial communities. The higher gas production in the cellulase-induced sample (*filled circle*) is simply due to higher organic load

In the cellulase induced batch sample, cellulolytic activity reached the detection limit after an incubation time of 48 h, increasing only slightly by day 3 but dramatically increasing by day 4, with a maximum signal for cellulase activity after 5 days (120 h, Fig. 1a). The activity thereafter decreased and was not detectable after 9 days. Contrary to this, the corresponding samples from the reference batch bottle, with glucose as the sole nutritional carbon source, did not show any increase in cellulase activity over the entire period (Fig. 1a). As noted earlier, the increase in cellulase activity in the filter paper-induced sample was preceded by yellow discoloration of the filter paper on day 3 [22], indicating the presence of the bacterial class clostridia, such as *Clostridium thermocellum* [33] or *Ruminococcus flavefaciens* [34]. Gas production commenced almost immediately, with no change in pH, after only a very short lag phase (Fig. 1b). Directly after the onset of gas production a difference in production rate could be detected, by which the cellulose containing sample showed a higher gas production rate over the experiment period, and higher final yield at the end of the experiment (Fig. 1b). At this time, it could also be concluded that the cellulose substrate was entirely consumed. On the final day of incubation, a methane content of 22% was registered in the head space of the induced sample as compared to 9% for the control. Samples selected for analysis and comparison of the protein expression pattern by 2-D DIGE were collected from the induced sample at 24 h (Ind_24 h_), when no cellulase activity was registered, and at 96 h (Ind_96 h_), at intermediate cellulase activity (Fig. 1a). At the same time point, a sample was withdrawn from the non-induced reference sample (Ref_96 h_). Notably, the amount of protein (dissolved in 500 μl 2-DE loading buffer) collected from 100 ml of cell suspension supernatant at 96 h was 2.7 times higher in the Ind_96_ sample (1.42 mg/ml) than in the Ref_96 h_ sample (0.52 mg/ml). For the Ind_24_ sample, the registered protein concentration was 0.74 mg/ml, corresponding to only half that of the Ind_96 h_ sample (Table 1).

**Table 1:** Detected protein spots and differences between samples

Sample	Amount (mg)^a^	Number of spots	Diff.	Match rate 1 (%)^b^	Match rate 2 (%)^c^	New or 2 times upregulated^d^	New or 2 times upregulated^e^
Ind_96 h_	1.42	525		99	58		
Ref_96 h_	0.74	484	41	97	53	62	21
Ind_24 h_	0.52	337	188	97	37	54	

^a^From 100 ml cell suspension supernatant concentrated to 10 ml

^b^Match rate 1 denotes the ability to find spots on the constructed master gel also in an individual gel and gives an indication of the quality of the gels and gel matching

^c^Match rate 2 denotes the percentage of matched spots on an individual gel relative to the total number of spots on the master gel

^d^Ind_96 h_ compared to the respective reference

^e^Ind_96 h_ compared to both references combined

### 2-D DIGE analysis and spot selection

**Fig. 2:**
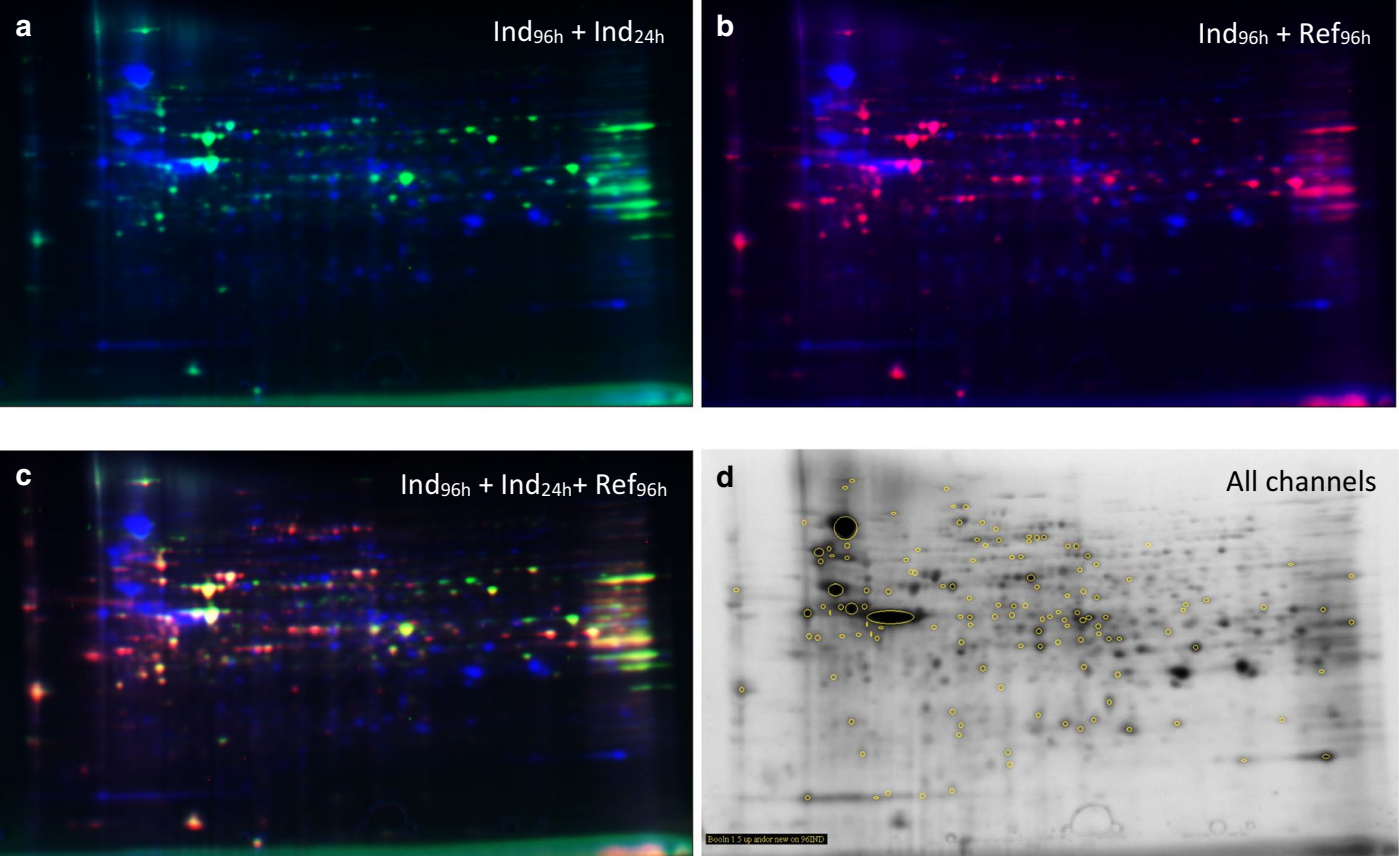
2-D DIGE gels. **a** The difference in protein expression pattern between the induced sample at 96 h (Ind_96 h_, *blue*) and induced sample at 24 h (Ind_24 h_, *green*). **b** The difference in protein expression pattern between the Ind_96 h_ sample and the non-induced reference sample at the same time point (Ref_96 h_, *magenta*). **c** Multichannel fluorescent view of the 2-D DIGE gel of all three variants. **d** All spots in the 2-D DIGE master gel

Sample preparation and CyDye labeling of each state resulted in high quality samples. This was confirmed by the well-separated protein spots in each channel, which were distributed over the full pH and size range of the 2-DE gel with very little background signal (Fig. 2a–d). The overall number of individual spots detected for each protein preparation from different samples differed significantly (Table 1). The highest number were detected in the Ind_96 h_ sample (525 spots); an intermediate number were detected in the Ref_96 h_ sample (484 spots) and the lowest number of spots in the Ind_24 h_ sample (337 spots).

To specifically identify proteins that differed over time and/or space, and thereby identify induced proteins, the expression pattern and expression levels of proteins in the Ind_96 h_ sample were compared to those for the Ind_24 h_ and Ref_96 h_ samples. It was found that 62 protein spots could be assigned as new or two times upregulated in the Ind_96 h_ sample as compared to the Ref_96 h_ sample (Table 1). However, in comparison to the Ind_24 h_ sample, only 54 protein spots were found to be new or upregulated in the Ind_96 h_ sample, even though the difference in the total number of protein spots was much larger for the Ind_96 h_ sample than the Ind_24 h_ sample (188 spots). Of all the proteins analyzed in the two comparisons, 21 new or two fold upregulated protein spots detected in the Ind_96 h_ sample were found to be common to both the Ind_24 h_ and Ref_96 h_ samples (Table 1).

### LC–MS/MS analysis and identification by database search

**Fig. 3:**
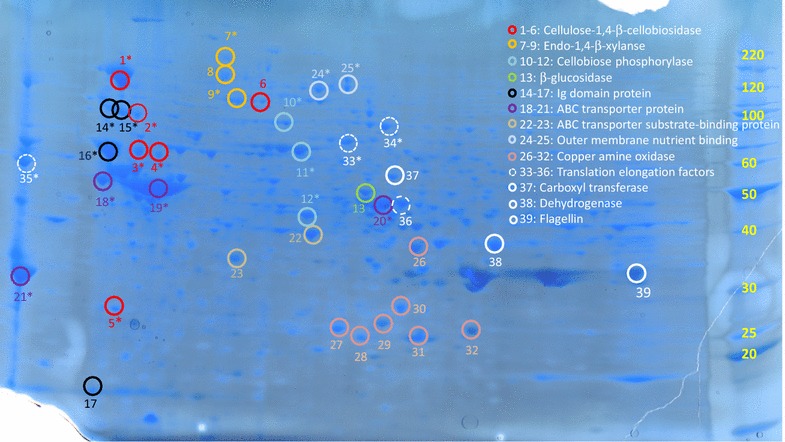
Preparative 2-D gel of the Ind_96 h_ sample for spot picking and tandem mass spectrometry of proteins identified as upregulated. Selected and analyzed spots are encircled and color coded for functional identification

From the preparative 2-D gel, 39 of the protein spots found to be most upregulated by 2-D DIGE were visually detected (Fig. 3) and excised for analysis by mass spectrometry. Based on the parameters and limitations described in the “Methods” section, the identified proteins were able to be assigned to a restricted number of functions and categories. Notably, 17 (44%) out of the 39 analyzed protein spots were assigned as carbohydrate-active enzymes (Table 2), while many of the remaining non-carbohydrate-active proteins were related to the metabolism of cellulose (Table 3).

**Table 2:** Upregulated protein spots identified as carbohydrate-active enzymes

Spot no.	Method	No. of peptides	Of which unique	Score (−10lgP)	Database entry by PEAKS DB and SPIDER	Name	Category^a^
1	PEAKS DB SPIDER	8	2	216.7	Gi|489608295^b^	Cellulose 1,4-β-cellobiosidase	CAZy GH 9, NC, SP (1–27)
		9	3	246.3			
2	PEAKS DB SPIDER	8	2	162.1	Gi|489608295^b^	Cellulose 1,4-β-cellobiosidase	CAZy GH 9, NC, SP (1–27)
		11	4	172.5			
3	PEAKS DB SPIDER	5	2	109.7	Gi|489608295^b^ Gi|740458809^c^	Cellulose 1,4-β-cellobiosidase	CAZy GH 9, NC, SP (1–27)
		13	8	148.5			
4	PEAKS DB SPIDER	4	2	108.6	Gi|489608295^b^ Gi|740458809^c^	Cellulose 1,4-β-cellobiosidase	CAZy GH 9, NC, SP (1–27)
		4	2	116.6			
5	PEAKS DB SPIDER	1	1	91.99	Gi|489608295^b^	Cellulose 1,4-β-cellobiosidase	CAZy GH 9, NC, SP (1–27)
		1	1	91.99			
6	PEAKS DB SPIDER	7	5	126.8	Gi|489614249^b^	Cellulose 1,4-β-cellobiosidase	CAZy GH 9, NC, SP (1–27)
		8	3	133.8			
7	PEAKS DB SPIDER	17	4	216.7	Gi|575088796^c^	Endo 1-4-β-xylanase	CAZy GH 11, NC, SP (1–29)
		34	14	246.3			
8	PEAKS DB SPIDER	4	2	121.9	Gi|489614855^b^ Gi|489609419^c^	Carbohydrate-binding protein Endo 1-4-β-xylanase precursor	CAZy GH 11, NC, SP (1–29)
		4	4	121.9			
9	PEAKS DB SPIDER	5	3	97.4	Gi|489614249^b^ Gi|575088796^c^	Cellulose 1,4-β-cellobiosidase Endo 1-4-β xylanase	CAZy GH 9, NC, SP (1–27) CAZy GH 11, NC, SP (1–29)
		6	3	106.1			
10	PEAKS DB SPIDER	8	2	149.6	Gi|575088170^c^	Cellobiose phosphorylase	CAZy GH 11, TM, SP (1–27)
		9	3	152.6			
11	PEAKS DB SPIDER	9	2	136.7	Gi|575088170^c^ Gi|489613736^b^	Cellobiose phosphorylase Glycosyl transferase	CAZy GH 94, TM
		9	2	139.2			
12	PEAKS DB SPIDER	2	1	74.76	Gi|740457862^c^	Glycosyl transferase	CAZy GH 94, TM
		2	1	74.76			
13	PEAKS DB SPIDER	7	2	120.6	Gi|489616357^b^	β-Glucosidase	CAZy GH 1, TM (weak), NC
		9	2	124.2			
14	PEAKS DB SPIDER	14	4	243.8	Gi|553726407^b^ Gi|489608943^b^	Hypothetical Ig domain protein	Carbohydrate act. enzyme domain, NC, SP (1–26)
		22	10	260.9			
15	PEAKS DB SPIDER	8	4	178.1	Gi|553726407^b^ Gi|489608943^b^	Hypothetical Ig domain protein	Carbohydrate act. enzyme domain, NC, SP (1–26)
		12	7	193.2			
16	PEAKS DB SPIDER	9	3	166.4	Gi|553726407^b^ Gi|489608943^b^	Hypothetical Ig domain protein	Carbohydrate act. enzyme domain, NC, SP (1–26)
		15	5	184.8			
17	PEAKS DB SPIDER	4	4	155.9	Gi:489613676^b^	Hypothetical Ig domain protein	Carbohydrate act. enzyme domain, NC, SP (1–26)
		4	4	155.9			

^a^In CAZy database; *GH* glycoside hydrolase family no., *NC* non-cellulosomal, *SP* signal peptide, signal peptide sequence length in brackets, *TM* transmembrane

^b^ *Ruminiclostridium thermocellum*

^c^ *Clostridium straminisolvens*

**Table 3:** Upregulated non-carbohydrate-active proteins/enzymes

Spot no.	Method	No. of peptides	Of which unique	Score (−10lgP)	Database entry by PEAKS DB and SPIDER	Name	Category^a^
18	PEAKS DB SPIDER	25	20	247.8	Gi|740455585^b^	ABC transporter	substrate binding protein, NC, SP (1–21)
		50	45	274.5			
19	PEAKS DB SPIDER	43	42	234.4	Gi|740455585^b^	ABC transporter	substrate binding protein, NC, SP (1–21)
		76	69	251.8			
20	PEAKS DB SPIDER	2	2	110.7	Gi|740455585^b^	ABC transporter	substrate binding protein, NC, SP (1–21)
		4	4	143.8			
21	PEAKS DB SPIDER	3	3	94.4	Gi|740455585^b^	ABC transporter	substrate binding protein, NC, SP (1–21)
		5	5	112.9			
22	PEAKS DB SPIDER	4	4	118.8	Gi|503373723^d^	ABC transporter substrate-binding	Solute-binding family 1, NC, SP (1–24)
		5	5	131.2			
23	PEAKS DB SPIDER	8	7	156.1	Gi|502813980^e^	ABC transporter substrate-binding	Solute-binding family 1, TM (weak), SP (1–27)
		8	7	156.1			
24	PEAKS DB SPIDER	17	16	197.0	Gi|697632338^f^	Putative outer membrane protein	Nutrient binding, NC, SP (1–30)
		20	18	203.3			
25	PEAKS DB SPIDER	10	7	209.0	Gi|697632338^f^	Putative outer membrane binding	Nutrient binding, NC, SP (1–30)
		12	8	214.2			
26	PEAKS DB SPIDER	2	2	56.3	Gi|489614243^b^ Gi|489608260^b^	Copper amine oxidase	NC, SP (1–32)
		2	2	56.3			
27	PEAKS DB SPIDER	13	13	174.0	Gi|489608992^b^	Copper amine oxidase	NC, SP (1–22)
		27	27	199.9			
28	PEAKS DB SPIDER	2	2	86.2	Gi|489608992^b^	Copper amine oxidase	NC, SP (1–22)
		6	6	120.3			
29	PEAKS DB SPIDER	9	1	155.7	Gi|489613949^b^	Copper amine oxidase	NC, SP (1–24)
		11	3	160.6			
30	PEAKS DB SPIDER	3	3	113.6	Gi|489613949^b^ Gi|500163071^b^	Copper amine oxidase	NC, SP (1–24)
		3	3	113.6			
31	PEAKS DB SPIDER	2	2	83.2	Gi|740459505^c^ Gi|553726325^b^	Copper amine oxidase	NC, SP (1–26)
		4	2	100.8			
32	PEAKS DB SPIDER	1	1	76.4	Gi|740459505^c^	Copper amine oxidase	NC, SP (1–26)
		2	2	94.4			
33	PEAKS DB SPIDER	12	2	184.3	Gi|575090155^c^	Translation elongation factor G	Protein synthesis
		14	2	186.9			
34	PEAKS DB SPIDER	29	4	167.3	Gi|489609866^b^ Gi|575090155^c^	Translation elongation factor G	Protein synthesis
		36	9	175.8			
35	PEAKS DB SPIDER	4	1	144.6	Gi|575090155^c^	Translation elongation factor G	Protein synthesis
		5	1	152.6			
36	PEAKS DB SPIDER	50	2	169.2	Gi|740455515^c^	Translation elongation factor Tu	Protein synthesis
		55	2	169.2			
37	PEAKS DB SPIDER	45	10	241.4	Gi|489611743^b^	Carboxyl transferase	Gluconeogenesis, TM (weak)
		48	11	244.5			
38	PEAKS DB SPIDER	56	9	278.6	Gi|740456462^b^	Glyceraldehyde-3-phosphate dehydrogenase	Glycolysis
		62	11	281.2			
39	PEAKS DB SPIDER	33	5	234.6	Gi|740455993^c^	Flagellin	Motility
		41	5	241.3			

^a^In CAZy database; *GH* glycoside hydrolase family no., *NC* non-cellulosomal, *SP* signal peptide, signal peptide sequence length in brackets, *TM* transmembrane

^b^ *Ruminiclostridium thermocellum*

^c^ *Clostridium straminisolvens*

^d^ *Sphaerochaeta globose*

^e^ *Aminobacterium colombiense*

^f^ *Fermentimonas caenicola*

Spots 1–6 and 9 all contained sequences of cellulose 1,4-β-cellobiosidases belonging to the CAZymes glycoside hydrolase (GH) family 9 of endo-cellulases, which hydrolyse the (1→4)-β-d-glucosidic linkages in cellulose and cellotetraose, releasing two-sugar cellobiose from the non-reducing end of the cellulose chain (Table 2). All these protein spots, except for a single fragment (spot 5), were clustered in the upper left corner of the 2-D gel (Fig. 3), indicating similar pI and molecular weight. PEAKS DB identification of all the proteins in spots 1–6 annotated them to *Ruminiclostridium thermocellum* with the same protein identifier for spots 1–5 (Gi|489608295) and a different protein identifier for spot 6 (Gi|489614249). This indicates the exact same protein was present in spots 1–5 despite the varying pI and molecular weight. Detection of the same protein may suggest fragmentation of the spot with the highest molecular weight (spot 1). However, it could also be that the enzyme function is part of proteins with different modular compositions with different pI and M_w_ and the protein in spot 1 is simply the largest of these different variants. A third alternative is of course that the different cellulose 1,4-β-cellobiosidases are all unique proteins with different pI and *M*_w_ but annotated as produced by *Ruminiclostridium thermocellum* because the actual producing microorganisms have not yet been sequenced and the correct enzyme entries are, thus, not available in the NCBI nr database. Notably, by using the SPIDER algorithm, which produces alternative peptide sequence tags, and thus compensates for de novo sequencing errors and homologous database entries, the cellobiosidases in spots 3 and 4 were annotated with a higher probability score as cellulose 1,4-β-cellobiosidases originating from *Clostridium straminisolvens*. Also, the protein in spot 9 was assigned by PEAKS DB as the CAZyme 1,4-β-cellobiosidase from *Ruminiclostridium thermocellum.* However, in this case, SPIDER identified a hit with a higher score for an enzyme not only of a different microbial origin but also of different function (endo 1-4-β xylanase). Spots 7–9 all contained endo 1-4-β xylanase if the algorithm producing the highest score (SPIDER) was assumed to provide the most correct result. Endo 1-4-β xylanases degrade the linear polysaccharide β-1,4-xylan into xylose, thus breaking down hemicellulose.

Spots 10–12 were identified as either cellobiose phosphorylase or glycosyl transferase, which both possess the same enzymatic activity. Glycoside phosphorylases catalyze cleavage of glycosidic bonds by substitution with phosphate and are named using the combination of the name of the substrate and the term “phosphorylase.” Thus, cellobiose phosphorylases use cellobiose (produced by cellulose 1,4-β-cellobiosidases) as substrate. In the literature, these enzymes are considered to be intracellular enzymes produced by anaerobic bacteria. In accordance, the genes indicated by the analysis were predicted to be coding for transmembrane proteins without a signal peptide for export. Interestingly, these enzymes have also attracted attention for use in the production of biofuels from plant biomass [35].

Spot 13 was identified as a β-glucosidase which hydrolyses glycosidic bonds in β-d-glucosides and oligosaccharides with the release of glucose. β-Glucosidases are key enzymes in the hydrolysis of cellulose because they complete the final step during cellulose hydrolysis by converting cellobiose to glucose. This reaction is a major bottleneck against the efficient biomass conversion of cellulose because it is always controlled and inhibited by its product glucose. Therefore, a significant challenge when attempting to improve the bioconversion of lignocellulosic biomass is to identify glucose tolerant β-glucosidases [36].

Spots 14–17 were all assigned as hypothetical Ig domain proteins. The spots were clustered in the same region as the cellulose 1,4-β-cellobiosidases (Fig. 3), except for spot 17, which was of lower molecular weight and most likely a peptide fragment. It can be assumed that these spots also contained cellulases, given that these proteins were strongly upregulated in response to cellulose and that it is known that many modular cellulases contain an Ig domain N-terminally fused to the catalytic domain of cellulases. These Ig domain containing cellulases are of considerable interest because the Ig domain is important for the cellulolytic activity and is believed to confer stability to the enzyme [37].

The remaining non-carbohydrate-active enzymes were identified as follows (Table 3). Spots 18–21 were all identified as ABC transporter proteins. ABC transporters all use ATP hydrolysis to pump molecules across membranes and cover a wide spectrum of substrates, including small organic compounds such as amino acids and sugars but also larger molecules such as peptides and polysaccharides. Given the high cellulolytic activity and the fact that 1 and 3% of bacterial and archaeal genomes encode subunits of ABC transporters, respectively [38], it was not unexpected to register upregulation of these proteins.

Spots 22 and 23 were identified as ABC transporter substrate-binding proteins. These are also part of the ABC transporter system as highly specific periplasmic solute-binding proteins. Notably, the identified proteins belonged to the solute-binding family 1, which include multiple oligosaccharide binding proteins (Interpro, 2016). Spots 24 and 25 were identified as two additional putative outer membrane nutrient binding proteins.

Spots 26–32 were all identified as copper amine oxidases. These enzymes catalyze the oxidation of primary amines to aldehydes with subsequent release of ammonia and hydrogen peroxide, requiring one copper ion per subunit and topaquinone as a cofactor [39]. In prokaryotes, their function is to enable various amine substrates to be used as sources of carbon and nitrogen. However, given that the only nutrient source in the induced sample was cellulose and that no proteases were identified as upregulated, it is intriguing that these copper amine oxidases were so obviously upregulated. Although it is speculative, it can be hypothesized that the copper amine oxidases may also play an auxiliary function in the degradation of lignocellulose. Recently, the CAZy database was expanded to include various auxiliary enzymes, of which many produce reactive hydrogen peroxide. These include, e.g., the auxiliary activities (AA) of family AA5_1 (glyoxal oxidase activity), AA3_2 (including aryl-alcohol oxidase and glucose 1-oxidase), and AA3_3 (alcohol oxidases), which all generate the H_2_O_2_ necessary for the function of class II peroxidases to perform the oxidation of lignin and for hydroxyl radical generation in Fenton reactions [40]. Notably, whereas glucose 1-oxidase is an intracellular fungal enzyme with no known export system for the generated H_2_O_2_, all the copper amine oxidases identified in spots 26–32 were non-cytosolic proteins equipped with a signal peptide. Furthermore, all the oxidizing AA enzymes in CAZy are of fungal origin. Thus, the copper amine oxidases identified in this work could be the first example of prokaryotic auxiliary H_2_O_2_-producing enzymes involved in lignocellulosic degradation. However, the oxidation reaction with amines requires oxygen, which is unavailable in strictly anaerobic environments. Notably though, the upregulation of these copper amine oxidases was not due to sample handling since they were not upregulated in the non-induced Ref_96 h_ sample treated in exactly the same way as the Ind_96 h_ sample except without the addition of the complex cellulose nutrient. Spots 33–38 were all identified as intracellular enzymes, including 4 translation elongation factors, indicating high protein synthesis activity, while the remaining two were linked to the metabolism of sugar, including a carboxyl transferase (gluconeogenesis) and a glyceraldehyde-3-phosphate dehydrogenase (glycolysis). The final protein identified in spot 39 was flagellin, which is involved in the motility of microorganisms.

In summary, of the 39 protein spots analyzed, 29 (74%) of the identified sequences were found to carry a signal peptide (SP) for export and 28 (72%) were predicted to be non-cytosolic (NC) proteins. Of the 28 proteins predicted as non-cytosolic, 10 (36%) belonged to carbohydrate-active enzymes (CAZymes). In addition, 4 (14%) of the non-cytosolic proteins were identified as Ig domain proteins (Table 2). Seven (25%) were found to be involved in nutrient binding and transport and 7 (25%) belonged to the group of copper amine oxidases (Table 3). Three proteins, all CAZyme cellobiose phosphorylases, were predicted to be transmembrane proteins (Table 2), whereas only five selected proteins were identified as intracellular (Table 3).

Notably, for the majority of the proteins (31 spots, or 79%), a larger number of peptide sequence tags were assigned to the identified sequence by SPIDER than by PEAKS DB (Tables 2, 3). In most cases, this simply strengthened identification of the database entry by adding peptide sequence tags to the ones generated by PEAKS (see, e.g., spot 1 in Table 2). However, in 10 cases, the additional sequence tags generated by SPIDER resulted in improved identification of an alternative protein with the same function but different database entry and better score (see, e.g., spot 3 in Table 2). In two cases, the additional peptide sequence tags produced by SPIDER identified an alternative protein with a different function than the one identified by PEAKS DB (see, e.g., spot 9 in Table 2). That is, by permitting alternative, including mutated, sequences of the de novo sequence tags generated by PEAKS, additional sequence tags from the mass data of the same protein spot were generated by which a better assigned, alternative or different protein was identified.

All identified proteins were annotated to originate from only five microbial species, including *Ruminiclostridium thermocellum*, *Clostridium straminisolvens*, *Sphaerochaeta globosa*, *Aminobacterium colombiense*, and *Fermentimonas caenicola*, all of which are strict or facultative anaerobes [41–45]. Interestingly, *Aminobacterium colombiense* is understood to not utilize carbohydrates such as glucose or cellobiose [44], although the identified oligosaccharide binding solute binding family 1 protein suggests otherwise (spot 23, Table 3).

## Discussion

### Enzyme induction, separation, and selection

The fast onset of biogas production and stable pH in the differential batch cultures indicated that the cells were undamaged by the treatment prior to the batch start. Thus, the samples contained a complete viable microbial community of primary fermenters, secondary fermenters and methanogens to digest, metabolize and mineralize the organic compounds to CO_2_ and CH_4_. As reported in earlier experiments [22], cellulase activity was distinctively induced from the enzyme suppressed state. In addition, the enzyme suppressed state was retained in the reference batch supplied with chemically defined medium. Thus, the dynamic changes in protein expression pattern due to cellulase induction could in theory be analyzed either over time in the same batch or over space against a reference batch (Fig. 1a). However, because the pelleted microbial community was washed before transfer to separate batches, much less protein was collected and fewer spots were identified in the Ind_24 h_ sample. This made the automated image analysis for new or upregulated proteins in the Ind_96 h_ sample over time problematic as it was difficult to match and compare the protein expression pattern between the two times. This was primarily because the total number of proteins differed significantly (by 188 spots), and therefore many candidate proteins were likely false positives. That is, many of the additional 188 protein spots registered in the Ind_96 h_ as compared to the Ind_24 h_ sample may have arisen from accumulation of constitutively expressed proteins and not necessarily been produced in response to the inducing substance. Thus, this comparison over time would be less stringent in terms of the goal to preferentially identify only the proteins that were new or upregulated in response to the inducing substance specifically. Thus, clearly, comparison over space against an identically treated reference, except for the single parameter of the inducing substance, is a better approach. Despite the fact that the Ind_96 h_ and Ref_96 h_ samples were incubated for the same time, a considerably larger amount of protein was prepared from the extracellular liquid of the Ind_96 h_ sample than from the Ref_96 h_ sample. This strongly indicates that the higher cellulolytic activity in the Ind_96_ sample was due to the presumably higher expression and secretion of a number of proteins linked to the degradation and metabolism of cellulose. This was evident in the value of match rate 2 of the identified spots (Table 1), i.e., Ind_96 h_ had a match rate of only 58% against the master gel. Thus, 42% of the detected spots identified in the master gel were derived from either the Ind_24 h_ or the Ref_96 h_ sample and not detected in the Ind_96 h_ sample despite the much higher amount of proteins collected from the cellulase-active Ind_96 h_ sample. This result stems from the fact that the protein complement in the Ind_96 h_ sample was dominated by proteins that were upregulated in response to the addition of cellulose. Thus, when the same amount of protein of each state (50 μg) was loaded onto the gel, less abundant proteins in the Ind_24 h_ or the Ref_96 h_ samples made up a larger fraction of the protein pool and were detectable. However, even when the same proteins were present in the Ind_96 h_ sample, these proteins constituted a much smaller part of the total protein pool in the Ind_96 h_ sample and were undetectable because the protein complement of the Ind_96 h_ sample was so heavily dominated by the upregulated proteins. In this sense, the strongly induced enzyme expression from an enzyme suppressed state was almost excessively efficient, resulting in a manifold increase in expression and activity of targeted enzymes (Fig. 1). Nevertheless, only proteins that were upregulated were of interest and a good comparison between the Ind_96 h_ and the Ref_96 h_ sample was possible.

By comparing the Ind_96 h_ sample against the Ref_96 h_ sample, it was found that the samples differed by only 41 proteins in total (Table 1), of which many were expected to be related to the single difference in nutritional state between the two samples. In this comparison, a total 62 proteins were found to be new (41) or two times upregulated (21). Notably, the absolute number of detected spots was very high for a gel-based metaproteomics study, with 525 and 484 spots detected from the extracellular milieu of the Ind_96 h_ and Ref_96 h_ samples, respectively. This can be compared to, e.g., studies of the intracellular metaproteome of methanogenic microbial communities from biogas reactors, in which normally 200–400 spots are detected by gel-based methods [46], despite the expectation that the intracellular proteome should constitute a much larger fraction of the total protein complement. Thus, importantly, by comparing the protein expression pattern of the induced sample with an identically treated but non-induced sample, proteins that were constitutively expressed (in Ref_96 h_) could be subtracted from the Ind_96 h_ sample. Hence, of the 525 protein spots identified in the Ind_96 h_ sample, the downstream analysis could be limited to only the proteins that differed in number or intensity between the two samples. Thereby, the selection of targeted proteins was based on upregulation rather than simple abundance, as is evident in Fig. 3. By this approach, the 39 upregulated proteins that were visually identified and collected from the preparative gel were biased to be a product of the microbial need to degrade, take up and metabolize cellulose and its breakdown products. Since 28 of these proteins were predicted to be non-cytosolic and carrying a signal peptide for export, it was further demonstrated that the majority of the proteins recognized as upregulated (in the extracellular environment) were also in fact of extracellular origin, representing proteins targeted for in this work and demonstrating the feasibility of the approach for bioprospecting extracellular enzymes.

### Protein functional identification

Using the applied approach, a large fraction of carbohydrate-active enzymes were selected and identified. With 17 out of the 39 selected proteins being carbohydrate-active enzymes (Table 1), this constituted a hit rate of 44%. If H_2_O_2_-producing copper amine oxidases were also included as involved in the degradation of cellulose, the number increased to 24, or 62%. This hit rate can be compared to that in metagenomics projects, where it has been estimated to be one active clone per 1204 clones (0.083%) [11]. Thus, the hit rate for the proteins identified as being upregulated and selected to be of interest in the present work was 550–747 times higher. It can further be compared to metaproteomic studies of other methanogenic communities treating lignocellulosic biomass, for which sometimes no cellulolytic enzymes are detected at all [46]. However, this is to be expected since the aim of almost all metaproteomic studies is to understand microbial ecology and physiology by investigating the intracellular protein complement. If proteins connected to the uptake and metabolism of sugars were also included, the number of proteins identified as linked to the effect of the addition of cellulase inducing filter paper would be even larger.

The confident identification of copper amino oxidases was an unexpected result. However, it demonstrates a significant advantage of using metaproteomics in comparison to, e.g., sequence or activity-based enzyme bioprospecting by metagenomics. In the latter two cases, only activities that are sought for can be identified and they are limited to sequence homologs of known sequences or for which activity assays are available. In contrast, by using induced differential metaproteomics as implemented in this study, all functions that are related to the degradation of the inducing substance will be registered because the selection and identification are based on changes in the expression of any protein in response to the inducing substance.

### Applicability for enzyme bioprospecting in microbial communities

By using the applied approach, a very high hit rate for the targeted enzymes was accomplished. However, for bioprospecting novel and applicable enzymes, a correct protein or gene sequence for the identified enzyme is needed for cloning and production. This applies especially if the identified protein is produced by a microorganism that cannot be obtained in pure culture and can thereby not be used for production of the enzyme. For specific enzymes of particular interest, and given that enough DNA sequence information is obtained, the correct or homologous genes to the protein identified can be picked up by PCR directly from the metagenome of the studied community [47]. However, because the purpose of combining metaproteomics with metagenomic data is to provide a direct link between the final phenotype and the genetic potential [10], it would be desirable to directly identify the correct full length genes for the potentially many enzyme activities of interest in bioprospecting efforts. In mass spectrometry, the correct full length protein sequence cannot be guaranteed, because there will always be sequence gaps between the identified peptide sequences, although estimates of correctness against a database entry can be made based on the number of sequence tags, unique sequence tags and scores. Other general problems are that the de novo sequence tags could be incorrect or that the analyzed protein could lack a counterpart in public databases. In addition, when handling data derived from mixed microbial communities, the complexity increases dramatically. It can be anticipated that such biological samples will contain many unknown but related proteins with small sequence differences to those available in databases, which are derived from a limited number of microorganisms. This gives rise to an elevated protein inference issue because peptide sequences could originate from different proteins of the same organism or from homologous proteins from different species or strains [48]. Furthermore, if the microbial community contains closely related species, these are likely to also produce these homologous proteins. Thus, a single protein spot excised from a 2-DE gel may contain several homologous proteins of the same pI and *M*_w_ but with slightly different sequences. To address these issues, the identification by de novo sequence tags produced by PEAKS was compared to the results obtained from SPIDER. If SPIDER produced a result with a higher score, this was used for identification instead.

In some cases, when the protein was better identified with SPIDER, the identification changed. The SPIDER algorithm tries to match the de novo sequence tags generated from the mass data by PEAKS to the database protein sequence entries, thereby allowing alterations, including mutations, in the de novo sequence tag to better match the database entries. As is evident in many cases in Tables 2 and 3 (column 3 and 4), SPIDER allowed for more de novo sequence tags to be identified. In some cases, this simply led to more sequence tags being identified in the same database entry (e.g., spot 1, Table 2). In other cases, an alternative database entry of the same protein function was better identified by these altered de novo SPIDER sequence tags (e.g., spot 3, Table 2). In a third case, SPIDER better identified a different database entry of different function (e.g., spot 9, Table 2). Therefore, a more thorough study of the de novo sequence tags of these three cases was performed.

In the first case (spot 1, Table 1), for which a higher −10lgP score of the same database entry was provided by SPIDER, it was found that all 6 non-unique and 2 unique peptides generated by PEAKS DB remained the same in the SPIDER results. Thus, SPIDER was simply able to add an additional sequence tag to provide a better score. This was the case in many identifications (e.g., spot 7 and 18) where many de novo sequence tags were used for confident protein identification. In the second case, by which an alternative protein of the same function was better identified by SPIDER (e.g., spot 3), it was found that the two non-unique peptides generated by PEAKS DB remained the same in the SPIDER results. However, neither of the two unique peptides were the same, which is reasonable as they should be unique to the identified entry in the entire database, and thereby by definition need to be different in order to identify two different database entries. This can be interpreted as non-unique sequences being part of common structurally conserved regions, which thereby define a class of proteins, whereas unique sequences better define specific database entries. Thus, since both database entries were identified, it could be suspected that protein spot 4 did in fact contain two closely related proteins. Therefore, the FASTA sequences of both entries were retrieved from NCBI and analyzed by the Compute pI/*M*_w_ tool on the ExPASy server. It was found that the two proteins had very similar calculated pI (4.87 and 4.83) and molecular weight (137,116 and 137,513 Da, respectively), which were not easily resolved by 2-D gel electrophoresis. Thus, it cannot be excluded that there were two closely related homologous proteins in spot 4. Notably, the *M*_w_ values were not in accordance with the position of spot 4 on the gel. This may be because the identified functions form part of a larger system of modular proteins and the computed *M*_w_ includes sequences, such as signal peptides, that might be trimmed off in the mature protein. In the third case (spot 9), by which an enzyme of different function was identified by SPIDER, none of the non-unique or unique peptide sequence tags were the same in the PEAKS DB and SPIDER identifications. In addition, the two entries differed in the computed molecular weights of 100,621 and 131,519 Da for the identified cellobiosidase and xylanase, respectively. Thus, it could not be decided which of the two hits were the more correct. SPIDER, by adjusting the PEAKS generated de novo sequence tags, may have provided many more hits. Reasonably, this implies that the mass data either contained many peptides with small sequencing errors or, alternatively, many peptides that were not completely homologous to the sequences in the NCBI nr database. Consequently, in cases where more hits were provided by SPIDER, it could be that SPIDER simply corrected a sequencing error. However, it could also be that the original PEAKS generated de novo sequence tag was in fact correct and that the protein in the selected spot was a novel enzyme with no counterpart with the exact same sequence in the database. In effect, since it was not possible to distinguish between the two alternatives, it was not possible to unambiguously conclude which of the two sequences was correct. Hence, in those cases, the identified protein was not able to be confidentially assigned to a specific microorganism.

## Conclusions

Clearly, all the results above were made possible solely by the approach to initially maintain the microbial community at an enzyme suppressed metabolic steady state on a chemically defined medium under constant and controlled conditions [22]. Thereby, the pronounced induction of desired enzyme activity from a low baseline level and sampling of the proteins in the extracellular environments were made possible [23]. Hence, comparison of the protein expression pattern of the extracellular proteins of the two samples, with distinct but limited differences, enabled the identification of almost exclusively proteins that were upregulated in response to the inducing substance. However, the above findings also demonstrate a critical limitation of applying metaproteomics for bioprospecting novel enzymes in microbial communities if the main objective is to clone, produce, and characterize the identified protein because in most cases, it is not possible to unambiguously link the identified protein to the correct complete gene or protein. Unless the de novo sequence tags completely cover the sequence of the data base entry, usually only the function of the protein is identified. Therefore, there would be little point in producing enzymes of either identified sequences under the assumption that it would represent the induced enzyme in the respective protein spot. Thus, to make the best use of the metaproteomics-guided selection, it needs to be complemented by genetic information on the specific microbial community to enable identification of the precisely correct gene coding for the identified protein.

## Abbreviations

SI: Système International
NmL: mL gas normalized to standard SI conditions
2-D: two-dimensional
CHAPS: 3-[(3-cholamidopropyl)dimethylammonio]-1-propanesulfonate
DTT: dithiothreitol
IEF: isoelectric focusing
SDS: sodium dodecyl sulfate
DIGE: difference gel electrophoresis
Cy-Dye: cyanine dyes
LC-MS: liquid chromatography mass spectrometry
LTQ: linear trap quadrupole
FTMS: Fourier transform mass spectrometry
CID: collision-induced dissociation
PTM: post-translational modification
FDR: false discovery rate
NCBI nr: National Center for Biotechnology Information non-redundant
ALC: average local confidence
GC-FID: gas chromatography-flame ionization detector
CAZy: carbohydrate-active enzyme
